# Quantitative bioassay to identify antimicrobial drugs through drug interaction fingerprint analysis

**DOI:** 10.1038/srep42644

**Published:** 2017-02-16

**Authors:** Zohar B. Weinstein, Muhammad H. Zaman

**Affiliations:** 1 Department of Pharmacology and Experimental Therapeutics, Boston University School of Medicine, Boston, MA 02118, USA; 2 Howard Hughes Medical Institute, Boston University, Boston, MA 02215, USA; 3 Department of Biomedical Engineering, Boston University, Boston, MA 02215, USA.

## Abstract

Drug interaction analysis, which reports the extent to which the presence of one drug affects the efficacy of another, is a powerful tool to select potent combinatorial therapies and predict connectivity between cellular components. Combinatorial effects of drug pairs often vary even for drugs with similar mechanism of actions. Therefore, drug interaction fingerprinting may be harnessed to differentiate drug identities. We developed a method to analyze drug interactions for the application of identifying active pharmaceutical ingredients, an essential step to assess drug quality. We developed a novel approach towards the identification of active pharmaceutical ingredients by comparing drug interaction fingerprint similarity metrics such as correlation and Euclidean distance. To expedite this method, we used bioluminescent *E. coli* in a simplified checkerboard assay to generate unique drug interaction fingerprints of antimicrobial drugs. Of 30 antibiotics studied, 29 could be identified based on their drug interaction fingerprints. We present drug interaction fingerprint analysis as a cheap, sensitive and quantitative method towards substandard and counterfeit drug detection.

Drug interactions for a given phenotype are defined as a combinatorial effect of two drugs that is different than expected^1^. Drug interactions may be described as positive or negative depending on whether relatively more or less drug, respectively, is required to achieve a particular phenotype compared to single agents^1^. Sensitive drug interaction testing is limited by the combinatorial explosion necessary to evaluate multiple doses of drugs. Traditional checkerboard testing involves isobologram analysis for a square matrix of increasing concentrations of two drugs on each axis. Berenbaum theorized a simplified method of testing for drug interactions in which approximately equi-inhibitory doses of two agents are combined, titrated and compared to single agent dose response curves^2^.

Drug interaction analysis is a powerful tool to select potent combinatorial therapies^3^, predict connectivity between cellular components^4^ and drug mechanism of action^5^. Drugs with similar mechanism of action tend to have similar but not identical drug interaction profiles^6^. For instance, Yeh *et al*. report that the two 30S ribosome inhibitors doxycycline and tetracycline cluster together in a network analysis based on their drug interaction profiles. However, amongst the 21 antibiotics they are tested against, they show unique interactions with ampicillin and chloramphenicol, suggesting these varied combinatorial responses may be used to create a unique interaction fingerprint for these agents. A study of antifungal drug interactions found that two ergosterol synthesis (ERG11) inhibitors fluconazole and miconazole varied in their tendency towards suppressive drug interactions^7^. This suggests that drug interaction fingerprinting may be possible for any active pharmaceutical ingredient (API) that imparts a quantifiable phenotype such as luminescence or growth inhibition.

A key feature of drug quality assessment is the detection of APIs. Current methods to detect APIs are of limited utility due to great expense (HPLC^8^, NMR^9^), or low sensitivity/specificity (thin layer chromatography and colorimetric assays) of detection systems^10^. These technologies are often of prohibitive cost, grid power and expertise to be of widespread use in low- and middle-income countries (LMICs). Biosensors may fill the gap for a fast drug detection system that transduces a change in growth phenotype to a specific change in luminescence output. Bacteria based biosensors are inherently reproducible and are low cost to maintain and propagate. The luminescence phenotype is especially desirable as it limits the possibility of false positives that may arise from contamination in assays evaluating turbidity. Previous bacterial biosensors have been used for the detection of antibiotic susceptibility in mycobacteria^11^ and in drug mechanism of action studies by pharmaceutical companies^12^.

Approximately 25% of medicines in LMICs are counterfeit or substandard, the majority of which are antimicrobial agents^13^. Substandard medicines may contain impurities such as synthetic byproducts and degradation products or the wrong dose of active ingredient^14^. A recent survey of thousands of antimalarials, anti-mycobacterials and other antibiotics found 40% of sampled drugs failed quality testing in some world regions^15^. This is of particular concern due to the high global burden of malaria and tuberculosis. Tuberculosis alone accounts for 1.5 million deaths annually^16^. Besides the immediate concerns about treatment failures, substandard medicines are also hypothesized to contribute to the worldwide trend toward antimicrobial resistance^17^,^18^. Successful treatment of tuberculosis requires months of medication, the mainstay of which is rifamycins (e.g., rifampicin, rifapentine, rifabutin), often in combination therapy with 2–3 other agents^19^. Multidrug-resistant and extensively drug-resistant tuberculosis in particular necessitate prolonged combination therapy with up to 5 medications^20^. In 2014, there were an estimated 9.6 million new cases of tuberculosis, and 190,000 deaths due to multi-drug resistant tuberculosis^16^. Novel means to address the issue of substandard medicines may therefore decrease the health and financial burden associated with long-term treatment of all forms of tuberculosis.

In this study, we report a simplified checkerboard assay to quantify drug interactions between pairs of compounds in *Escherichia coli* expressing luciferase to expedite experiments. This methodology combines the sensitivity of larger checkerboard assays^1^ to identify cellular response at varying levels of inhibition with the ease of setup of high throughput 2 × 2 drug interaction matrices by the Bliss independence model, which is a multiplicative model assuming drugs act independently. Using this method, we are able to generate unique profiles of bacterial response to varying combinations of drugs to create unique fingerprints for four anti-mycobacterial agents and a major rifampicin degradation product. By comparing drug interaction profile similarity metrics, we developed a novel approach towards the identification of APIs.

## Results

### Drug interaction profiling from a systematic screen of 25 antibiotics in *E. coli*

**Figure 1:**
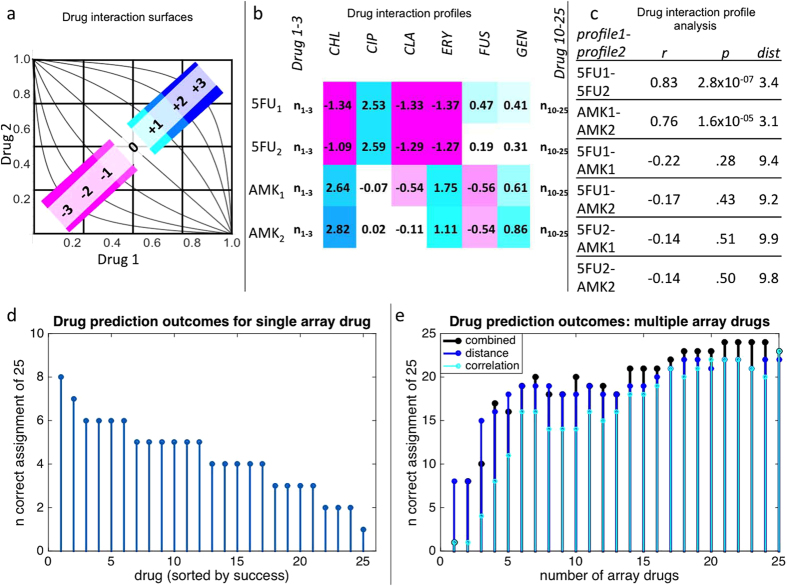
Drug interaction profile based identification of antibiotics. We first analyzed all pairwise interactions among 25 antibacterial drugs in *E. coli* for unique drug interaction profiles^21^. Drug interactions were approximated by the concavity of isophenotypic contours in a 2D grid of linearly increasing drug concentrations on each axis (**a**). Positive interactions are represented in blue, negative in magenta. A subset of interaction score replicates for query drugs, 5-fluorouracil and amikacin tested with array drugs chloramphenicol, ciprofloxacin, clarithromycin, erythromycin, fusidic acid and gentamicin (**b**). Drug interaction profiles are defined as a series of drug interaction scores for each query drug tested against a set of array drugs. Drug interaction profiles can be utilized for drug detection systems if the correlation of drug interaction profiles is greater for replicates than for comparison to other drug profiles (**c**). Alternatively, profile similarity may be based on minimum Euclidean distance between vectors of interaction. Euclidean distance between randomized replicates for query drugs against a single array drug could accurately identify 8/25 query drugs (**d**). Drugs were ranked based on their single agent identification value, serially added to the profile array and assessed for identification value based on Euclidean distance and/or rank correlation of profiles (**e**). Using the entire dataset, Euclidean distance, rank correlation and combined data could be used to correctly identify the vast majority of query drugs (22, 23, 24 correctly identified of 25, respectively).

In order to determine the feasibility of drug interaction profiling for API identification, we first analyzed a previously published dataset and unpublished data (literature set) of all pairwise interactions among 25 antibacterial drugs in *E. coli* for unique drug interaction profiles^21^. Figure 1a illustrates the experimental and analytical system for assessing drug interactions with the checkerboard method. In this paradigm, drug interactions are scored based on the concavity of isophenotypic contours. Concavity is determined by the logit function: log(x/(1 − x)) − log(y/(1 − y)); where x and y are normalized drug concentrations to achieve a similar level of inhibition. Figure 1b shows a subset of interaction scores for two drugs tested against a panel of 25 antibiotics in replicate. Drug interaction fingerprints (or profiles) are defined as a series of drug interaction scores for each query drug tested against a set of array drugs. Drug interaction fingerprints can be utilized for drug identification if the same drug tested against an array of other drugs is more similar to biological replicates than to the profile of other drugs. We used Spearman’s correlation and Euclidean distance between profiles as a metric of similarity (Fig. 1c).

To account for systematic experimental biases, we created 1,000 sets of profiles (25 × 25 × 1000, per set) with randomized replicate order from the literature set. The randomized profiles were then compared to identify drug identity based on minimal Euclidean distance and maximum correlation between interaction scores in replicate 2 vs. replicate 1, with each agent’s replicate 1 interaction score iteratively compared to replicate 2 scores of all 25 other agents. The most frequent replicate 2 ‘match’ of 1000 randomizations was compared to replicate 1 identity to assess successful identification of API. Distance metrics alone could identify up to 8/25 antibiotics using drug interaction scores with a single drug partner (Fig. 1d). Kanamycin, fusidic acid and erythromycin had the greatest single agent identification value (8, 7, 6 correct identifications, respectively). Starting with kanamycin, each of the remaining drugs was iteratively added to the analysis based on rank of single drug identification success. With all agents considered, 22/25 drugs were identified by distance alone (Fig. 1e). Considering the entire dataset, the Spearman’s correlation between replicate profiles successfully identified 23/25 agents. A combined analysis of correlation and distance scores allowed the successful identification of all but one of the 25 drugs (spectinomycin incorrectly identified as chloramphenicol). However, spectinomycin ranked as the second most likely agent by these metrics.

### A simplified sensitive method to assess drug interactions

**Figure 2:**
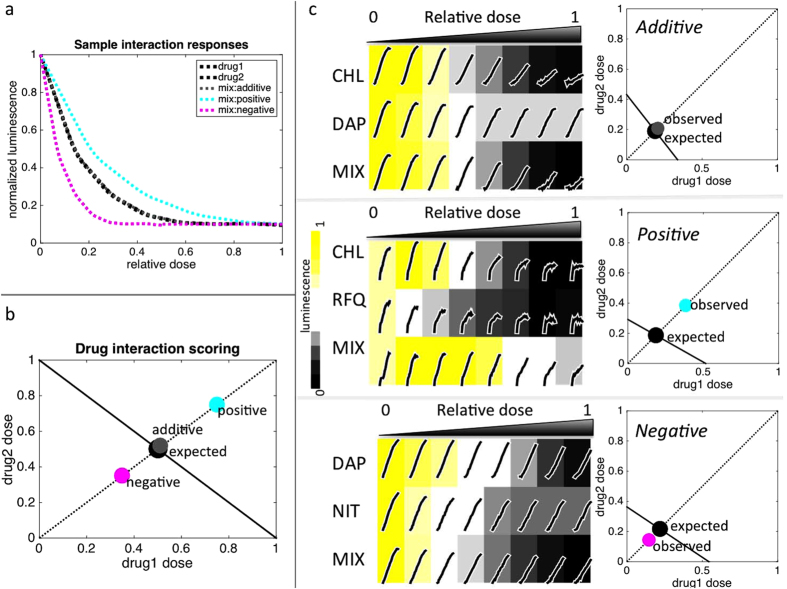
An expedited approach to drug interaction testing. We used a simplified, yet sensitive, approach to the classic 8 × 8 checkerboard method of drug interaction by sampling 24 concentrations of single or combined drugs for inhibition of *E. coli* luminescence (**a**). Drug interactions were assessed based on the amount of drug mixture (1/2 each drug 1 and drug 2, relative to individual drug dose) required to achieve the same inhibitory level as the constituent drugs^2^ for 40% inhibition of luminescence (IC40). Doses are plotted in Cartesian coordinates with the x and y intercepts set to drug 1 and drug 2 IC40; the intersection of the line y = x and that connecting the two intercepts defines the expected IC40 of the combination (**b**). The interaction score is defined as log_2_(observed/expected distance from the origin). Deviations are classified as negative or positive interactions. Representative experiments for additive, positive and negative interactions (**c** upper, middle and lower panels, respectively). At left are plots of raw RLU over time superimposed on a heatmap of luminescence output. Luminescence output was defined as the area under the RLU curve normalized to the no drug condition. At right are plots illustrating expected (black dot) and observed (grey, cyan and magenta dots) doses of drug mixture required to achieve 40% inhibition of luminescence, based on drug 1 and drug 2 IC40 levels. CHL = chloramphenicol, DAP = dapsone, NIT = nitrofurantoin, RFQ = rifampicin quinone, MIX = mixture of drug 1 and drug 2.

To expedite the construction of novel drug interaction profiles, drugs were evaluated based on the inhibition of luminescence of a constitutively expressed luciferase plasmid in *E. coli*. We used a simplified approach to the classic 8 × 8 checkerboard method of drug interaction by sampling 24 concentrations of single or combined drugs (Fig. 2a). An interaction score was computed based on the amount of drug mixture required to achieve 40% inhibition of luminescence compared to constituent drugs as single agents: log_2_(observed/expected dose) (Fig. 2b). Positive and negative interactions indicate that relatively more or less drug respectively was required for the combination to achieve the same level of inhibition than expected based on constituent drugs and a score of zero indicates no interaction (Fig. 2c).

**Figure 3:**
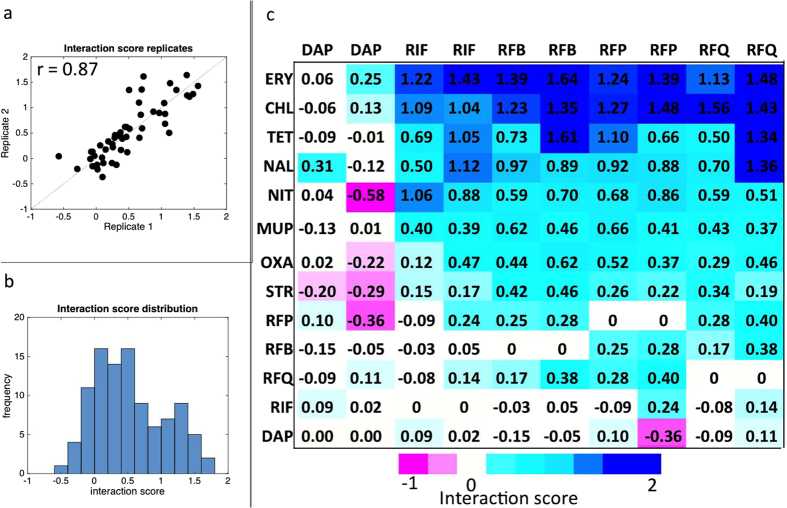
Interactions of pairwise combinations of anti-mycobacterial and antibiotic drugs are highly reproducible. Interaction score replicates for 50 drug pairs were highly reproducible (Spearman’s r = 0.87, p = 5 × 10^−16^) (**a**) and normally distributed based on the one-sample Kolmogorov-Smirnov test (**b**). The mean interaction score of all tested drug pairs (**c**). Clustering analysis reveals that dapsone had the most distinct interaction profile of all the query drugs, and rifapentine and rifabutin were most similar to each other. Of the rifampicin-related compounds, rifampicin quinone had the least similar correlation of interaction profiles, suggesting that drug interaction profiling may be used to differentiate rifampicin from its oxidation product.

We validated our method by assessing the pairwise interactions between 5 query drugs and 8 array drugs. The 5 query drugs contained 4 anti-mycobacterial agents [dapsone, rifampicin, rifabutin, rifapentine] and one major degradation product of rifampicin [rifampicin quinone]. The array drugs consisted of antibiotics of varying mechanisms of action [chloramphenicol, erythromycin, mupirocin, nalidixic acid, nitrofurantoin, oxacillin, streptomycin, and tetracycline]. The query drugs were selected based on their significance in tuberculosis treatment; array drugs were selected to provide a diverse range of interactions. All query drugs were also tested in combination with each other. Interaction score biological replicates were highly reproducible (Spearman’s r = 0.87, p = 5 × 10^−16^) (Fig. 3a) and normally distributed (Fig. 3b). Clustering analysis revealed that the rifampin related agents and degradation products had similar interaction profiles compared to the anti-folate agent dapsone (Fig. 3c). Rifampin-related drugs were enriched for antagonistic interactions (mean interaction scores, 0.47–0.58) while dapsone was more likely to have negative or null interactions (mean interaction score, 0).

### Drug interaction profiling of anti-mycobacterial agents in *E. coli*

**Figure 4:**
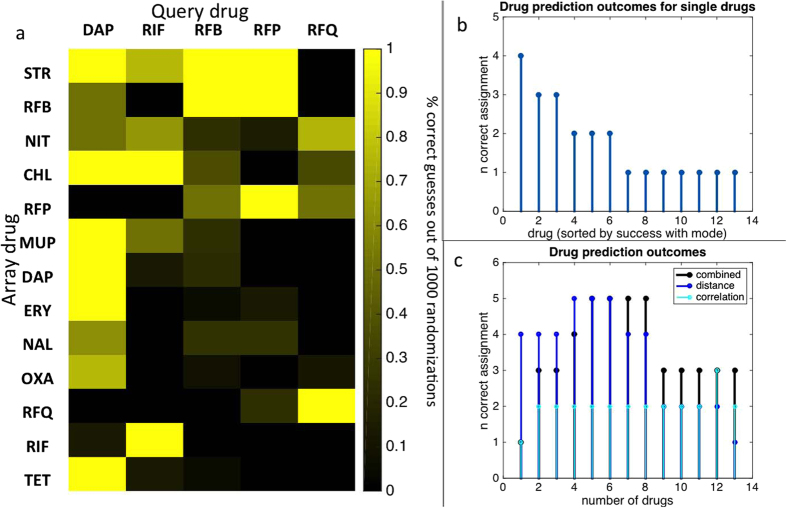
Drug identification success among the bioluminescent set of anti-mycobacterial agents. Drug profile similarity was assessed by examining correlation and distance between 1,000 sets of randomized profile replicates of the bioluminescent set. Array drugs varied greatly in their identification power for query drugs (**a)**. Euclidean distance between randomized replicates for query drugs against a single array drug could accurately identify 4/5 query drugs (**b**) based on interactions with streptomycin alone. Drugs were ranked based on their single agent identification value, serially added to the profile array and assessed for identification value based on Euclidean distance and/or rank correlation of profiles (**c**). Using the entire dataset, Euclidean distance, rank correlation and combined data could be used to correctly identify all query drugs. Using more than 8 array drugs in the analysis weakened the number of correctly assigned query drugs.

In accordance with the literature set, drug profile similarity of the bioluminescent set was initially assessed by examining correlation and distance between 1,000 sets of randomized profile replicates (13 × 5 × 1000, per set). Array drugs varied greatly in their identification power for query drugs (Fig. 4a). When only single drug interaction scores were considered among the profile, streptomycin was the drug with the best identification power (4/5 drugs identified accurately for the majority of 1,000 randomizations). Nitrofurantoin and rifabutin had the next best identification power (3/5 drugs each). The remainder of agents could be used to accurately identify up to 2 of the 5 query drugs (Fig. 4b). Dapsone, the only non-rifamycin agent tested, was unsurprisingly the simplest of the five query drugs to identify with 10/13 array drugs correctly identifying dapsone as single agents. For the majority of cases the rifampicin related drugs could be identified with 3–4 array drug data. In all, 4 array drugs were required to accurately discriminate between all 5 query drugs (Fig. 4c). Perhaps due to the close relationship between the query drugs, correlation scores could at best identify 3/5 query drugs and weakened the identification overall when combined with distance scores.

## Discussion

The World Health Organization has increasingly recognized the role of counterfeit and substandard medicines in the emergence and spread of antimicrobial resistant diseases^22^. Assuring drug quality of anti-mycobacterial agents has the potential to improve outcomes for the millions of people suffering from tuberculosis in LMICs worldwide. The current challenges in cost, training and infrastructure to assess drug quality requires novel approaches to identifying active pharmaceutical ingredients. Here, we utilized differential responses to drug combinations to create unique fingerprints for anti-mycobacterial drugs.

We used bioluminescent *E. coli* to assess drug interactions between pairwise combinations of 5 anti-mycobacterial agents and 8 additional antibacterial drugs for a total of 50 drug pairs in replicate. Overall, this expedited approach to drug interaction testing was highly reproducible, retaining the ease of experimental setup normally associated with 2 × 2 dose combination matrices^6^, while providing data for multiple levels of inhibition classically observable in checkerboard assays^23^. The use of luminescence rather than turbidity as a proxy for cellular growth reduces experimental runtime from the typical 12–24 hours to one hour, which is more amenable to the quick assessment of drug quality.

Biosensors have previously been shown to relay broad drug mechanism of action such as nucleic acid, cell wall or protein inhibition using luciferase and GFP reporters for drug target expression^12^,^24^. These biosensors have utility in drug discovery and environmental toxicology, but cannot distinguish between drugs with the same target. Drug interaction profiling can further narrow drug mechanism as drugs within the same class tended to cluster together, and unique drug interactions within classes can distinguish individual compounds.

This technique should extend to any type of drug, so long as it elicits a quantifiable phenotype, such as luminescence. Although *E. coli* is in the phylum of Proteobacter while mycobacterium is in the phylum Actinobacteria, there is a large overlap in anti-mycobacterial agents and drugs that inhibit the growth of *E. coli*. In particular, RNA polymerase inhibiting drugs such as rifampicin, rifabutin and rifapentine are generally broad-spectrum antibiotics and therefore effective across a wide range of bacterial species^25^,^26^. In our assay, *E. coli* are acting as a sensor and a one to one biological equivalent to mycobacteria is not essential for its utility in differentiating antimicrobial agents. However, not all anti-mycobacterial compounds affect *E. coli* growth. In such cases, the model organism *M. smegmatis* could be an alternative bacterium for creating drug interaction fingerprints^27^.

The analyses above suggest that interaction testing with only four array drugs are required to successfully identify the majority of query drugs from the literature and bioluminescent sets. Therefore, several unknown compounds could be identified on a single 96-well plate of interaction assays. Of all thirty query drugs evaluated in the literature and bioluminescent sets, only one could not be accurately identified based on the analytical methods described herein. The misattribution of chloramphenicol as spectinomycin suggests a high similarity between these two ribosome inhibitors. Our method therefore allowed for near complete identification of a large subset of antimicrobial agents, even for highly similar agents such as rifampicin, rifabutin and rifapentine. Furthermore, this method could differentiate rifampicin from rifampicin quinone, which indicates that drug interaction profiling can be used to detect the presence of drug degradation products.

A potential drawback of this approach may be the complexity of analyzing large drug-interaction datasets. This study used technologies such as a spectrophotometer and Matlab software for sensitive assessment of drug interactions. To convert this to a field test, photographic film may be used in lieu of a spectrophotometer to assess luminescence levels over time, and visual inspection can be used to verify if the drug interaction pattern matches the control drug. Alternatively, this assay could be adapted to a microfluidics based platform such as a lab in a suitcase (e.g., PharmaChk), which translates luminescence signal intensity to active pharmaceutical ingredient concentration^28^.

While this study evaluated 100 drug interactions overall to find unique drug interaction patterns among 5 query drugs, we found that as few as 4 array drugs are needed to differentiate even the highly similar rifamycin related compounds. Currently, there are approximately one hundred antibiotics that are in clinical use. Our study presented a quantitative bioassay to identify 30 antimicrobial drugs, which is a large subset of available medicines. Further studies could expand the repertoire of drug fingerprints, with prioritization given to other essential medicines that are most likely to be counterfeit or substandard. Thus, quantitative drug interaction profiling has the capacity to transform ongoing efforts to reduce the harm due to substandard drugs.

Systematic drug interaction profiling has many potential applications in drug discovery, mechanism of action and underlying cellular component connectivity. This manuscript presents the possibility of utilizing new drug interaction fingerprints in order to improve drug identification with applications in the detection of substandard and counterfeit medicines. Compared to the gold standard detection system of HPLC, our approach presents a fast, inexpensive and scalable method to assess drug quality.

## Materials and Methods

### Experimental conditions

Experiments were conducted with wild-type strain *E. coli* K12 strain DL41 with luciferase expressing SC101 plasmid^29^. Luciferase expression showed that luminescence output is highly reproducible. Replicates of area under the luminescence curve were highly correlated (r = 0.99, p = 2 × 10^−16^) and well correlated with inoculum density (r = 0.97, p = 8 × 10^−7^) (data not shown). All drugs were dissolved in DMSO and stored at −20 °C. Bacterial cells were grown in LB liquid culture overnight and diluted 1/100 and incubated at 37 °C for 1 hour before final plating on 96-well plates at a final OD_600_ of 0.05 in LB with the desired drug concentrations controlled for final solvent concentration of 2% DMSO. Plates were incubated at 37 °C in a temperature controlled microplate reader; with luminescence readings every 5 min. The following drugs were used in this study: chloramphenicol(CHL), dapsone (DAP), erythromycin (ERY), mupirocin (MUP), nalidixic acid (NAL), nitrofurantoin (NIT), oxacillin (OXA), rifabutin (RFB), rifapentine (RFP), rifampicin (RIF), rifampicin quinone (RFQ), streptomycin (STR), tetracycline (TET).

### Drug interaction metrics

Dose response was assessed for each single drug and combination (a one to one mixture) with linear dilutions. Drug combinations are an equal mixture of single agents containing approximately one half minimal inhibitory concentration of each single agent. Dose-response curves were generated based on the area under the luminescence curve, standardized to the drug free condition, over a one-hour interval. All doses are adjusted to fraction of minimal inhibitory concentration from zero to one.

In Cartesian coordinates, the x and y intercepts are set to the concentration of drug 1 and drug 2 to reach 40% inhibition of luminescence. The interaction score is defined as log_2_(observed/expected dose) along the y = x axis; where the expected is determined by the intersection of y = x with the line connecting the x and y intercepts and the observed is the dose of the drug mixture required to reach 40% inhibition of luminescence (Fig. 2b). The same drug combined with itself is assumed to have zero interaction.

### Drug identification metrics

Drug interaction score replicates were randomized to generate 1000 sets of replicates in order to limit systematic experimental bias in the data (Supplementary Figure 1). This translates to 2 sets of matrices of 25 query drugs × 25 array drugs × 1000 randomizations for the literature set, and 2 sets of matrices of 5 query drugs × 13 array drugs × 1000 randomizations for the bioluminescent set. The Euclidean distance from each row of query drug data from set 1 to all other rows from set 2 was iteratively determined for all 1000 randomizations. Overall, this generated a matrix of 25 × 25 × 1000 comparisons of distance scores for the literature set and 5 × 5 × 1000 comparisons for the bioluminescent set. A correct identification of the query drug was considered when the minimum Euclidean distance between each row in set 1 corresponded to the same row in set 2 (for example, rifabutin replicate 1 to rifabutin replicate 2). The same randomized datasets were assessed for the Spearman’s correlation between each row of set 1 and all rows in set 2. In this setup, a correct identification of the query drug was considered when the maximum correlation between each row in set 1 corresponded to the same row in set 2 (for example, rifapentine replicate 1 to rifapentine replicate 2). Overall prediction success was determined by whether or not the correct query drug was identified by the mode of 1000 predictions for Euclidean distance and Spearman’s correlation, respectively.

For the combined analysis, drug interaction score replicates were randomized to generate 1000 sets of replicates to limit systematic experimental bias in the replicates. Euclidean distance and Spearman’s correlation were determined between all query drugs across replicates for all 1000 sets. For each set of replicates, the drug identification algorithm identified the query drug from replicate 1 with the minimum Euclidean distance as well as the maximum correlation with the query drug from replicate 2, for a total of 2000 predictions overall. A successful identification was based on whether the correct drug was selected by the mode of the 2000 predictions.

## Additional Information

**How to cite this article**: Weinstein, Z. B. and Zaman, M. H. Quantitative bioassay to identify antimicrobial drugs through drug interaction fingerprint analysis. *Sci. Rep.* **7**, 42644; doi: 10.1038/srep42644 (2017).

**Publisher's note:** Springer Nature remains neutral with regard to jurisdictional claims in published maps and institutional affiliations.

## Supplementary Material

Supplementary Information

## References

[b1] Greco W. R., Bravo G. & Parsons J. C. The search for synergy: a critical review from a response surface perspective. Pharmacol. Rev. 47, 331–385 (1995). 7568331

[b2] Berenbaum M. C. A method for testing for synergy with any number of agents. J. Infect. Dis. 137, 122–130 (1978). 627734 10.1093/infdis/137.2.122

[b3] Zimmermann G. R., Lehár J. & Keith C. T. Multi-target therapeutics: when the whole is greater than the sum of the parts. Drug Discov. Today 12, 34–42 (2007). 17198971 10.1016/j.drudis.2006.11.008

[b4] Lehár J. . Chemical combination effects predict connectivity in biological systems. Mol. Syst. Biol. 3, 80 (2007). 17332758 10.1038/msb4100116 PMC1828746

[b5] Farha M. A. . Antagonism screen for inhibitors of bacterial cell wall biogenesis uncovers an inhibitor of undecaprenyl diphosphate synthase. Proc. Natl. Acad. Sci. USA 112, 11048–11053 (2015). 26283394 10.1073/pnas.1511751112 PMC4568241

[b6] Yeh P., Tschumi A. I. & Kishony R. Functional classification of drugs by properties of their pairwise interactions. Nat. Genet. 38, 489–494 (2006). 16550172 10.1038/ng1755

[b7] Cokol M. . Large-scale identification and analysis of suppressive drug interactions. Chem. Biol. 21, 541–551 (2014). 24704506 10.1016/j.chembiol.2014.02.012 PMC4281482

[b8] Liu J., Sun J., Zhang W., Gao K. & He Z. HPLC determination of rifampicin and related compounds in pharmaceuticals using monolithic column. J. Pharm. Biomed. Anal. 46, 405–409 (2008). 18055155 10.1016/j.jpba.2007.10.025

[b9] Salem A. A., Mossa H. A. & Barsoum B. N. Quantitative determinations of levofloxacin and rifampicin in pharmaceutical and urine samples using nuclear magnetic resonance spectroscopy. Spectrochim. Acta A Mol. Biomol. Spectrosc. 62, 466–472 (2005). 16257748 10.1016/j.saa.2005.01.016

[b10] World Health Organization (WHO). Survey of the quality of anti-tuberculosis medicines circulating in selected newly independent states of the former Soviet Union. (2013).

[b11] Riska P. F. . Rapid film-based determination of antibiotic susceptibilities of Mycobacterium tuberculosis strains by using a luciferase reporter phage and the Bronx Box. J. Clin. Microbiol. 37, 1144–1149 (1999). 10074539 10.1128/jcm.37.4.1144-1149.1999 PMC88662

[b12] Urban A., Eckermann S., Fast B. & Metzger S. Novel whole-cell antibiotic biosensors for compound discovery. Appl. Environ. Microbiol. 20, 6436–6443 (2007). 10.1128/AEM.00586-07 PMC2075059 17720843

[b13] Pincock S. WHO tries to tackle problem of counterfeit medicines in Asia. BMJ 327, 1126 (2003). 10.1136/bmj.327.7424.1126-a PMC1126859 14615319

[b14] Johnston A. & Holt D. W. Substandard drugs: a potential crisis for public health. Br J Clin Pharmacol 78, 218–243 (2014). 24286459 10.1111/bcp.12298 PMC4137817

[b15] Nayyar G. M. L., Breman J. G. & Herrington J. E. The global pandemic of falsified medicines: laboratory and field innovations and policy perspectives. Am. J. Trop. Med. Hyg. 92, 2–7 (2015). 25897072 10.4269/ajtmh.15-0221 PMC4455081

[b16] World Health Organization. Global tuberculosis report 2015. (2015).

[b17] Nayyar G. M. L., Breman J. G., Newton P. N. & Herrington J. Poor-quality antimalarial drugs in southeast Asia and sub-Saharan Africa. Lancet Infect. Dis. 12, 488–496 (2012). 22632187 10.1016/S1473-3099(12)70064-6

[b18] Baker S. Infectious disease. A return to the pre-antimicrobial era? Science 347, 1064–1066 (2015). 25745145 10.1126/science.aaa2868

[b19] Horsburgh C. R., Barry C. E. & Lange C. Treatment of Tuberculosis. N. Engl. J. Med. 373, 2149–2160 (2015). 26605929 10.1056/NEJMra1413919

[b20] World Health Organization. Companion handbook to the WHO guidelines for the programmatic management of drug-resistant tuberculosis. (2014). 25320836

[b21] Chandrasekaran S. . Chemogenomics and orthology-based design of antibiotic combination therapies. Mol. Syst. Biol. 12, 872 (2016). 27222539 10.15252/msb.20156777 PMC5289223

[b22] World Health Organization. The evolving threat of antimicrobial resistance: options for action: executive summary. (2012).

[b23] Cokol M. . Systematic exploration of synergistic drug pairs. Mol. Syst. Biol. 7, 544 (2011). 22068327 10.1038/msb.2011.71 PMC3261710

[b24] Hong H. & Park W. TetR repressor-based bioreporters for the detection of doxycycline using Escherichia coli and Acinetobacter oleivorans. Appl. Microbiol. Biotechnol. 98, 5039–5050 (2014). 24504461 10.1007/s00253-014-5566-1

[b25] Zenkin N., Kulbachinskiy A., Bass I. & Nikiforov V. Different rifampin sensitivities of Escherichia coli and Mycobacterium tuberculosis RNA polymerases are not explained by the difference in the beta-subunit rifampin regions I and II. Antimicrob. Agents Chemother. 4, 1587–90 (2005). 10.1128/AAC.49.4.1587-1590.2005 PMC1068591 15793146

[b26] Xu M., Zhou Y. N., Goldstein B. P. & Jin D. J. Cross-resistance of Escherichia coli RNA polymerases conferring rifampin resistance to different antibiotics. J. Bacteriol. 8, 2783–92 (2005). 10.1128/JB.187.8.2783-2792.2005 PMC1070395 15805525

[b27] Gordon S., Parish T., Roberts I. S. & Andrew P. W. The application of luciferase as a reporter of environmental regulation of gene expression in mycobacteria. Lett. Appl. Microbiol. 5, 336–40 (1994). 10.1111/j.1472-765x.1994.tb00469.x 7765445

[b28] Ho N. T., Desai D. & Zaman M. H. Rapid and specific drug quality testing assay for artemisinin and its derivatives using a luminescent reaction and novel microfluidic technology. Am J Trop Med Hyg. 6, S24–30 (2015). 10.4269/ajtmh.14-0392 PMC4455072 25897061

[b29] Kishony R. & Leibler S. Environmental stresses can alleviate the average deleterious effect of mutations. J. Biol. 2, 14 (2003). 12775217 10.1186/1475-4924-2-14 PMC193686

