# Supplementary material for: Quantitative bioassay to identify antimicrobial drugs through drug interaction fingerprint analysis
Source: Sci Rep. 2017 Feb 16;7:42644. doi: 10.1038/srep42644 (PMC5311984; doi:10.1038/srep42644)
Supplement: Supplementary Information [file srep42644-s1.pdf]

Supplementary Information to:

Quantitative bioassay to identify antimicrobial drugs through drug interaction fingerprint analysis

Zohar B. Weinstein[a] and Muhammad H. Zaman[b,c,*]

[a]Department of Pharmacology and Experimental Therapeutics, Boston University School of Medicine, Boston, MA 02118
[b]Howard Hughes Medical Institute, Boston University, Boston, MA 02215
[c]Department of Biomedical Engineering, Boston University, Boston, MA 02215

*Address correspondence to
Muhammad H. Zaman, zaman@bu.edu
Department of Biomedical Engineering
Boston University
44 Cummington Mall
Boston, MA 02215

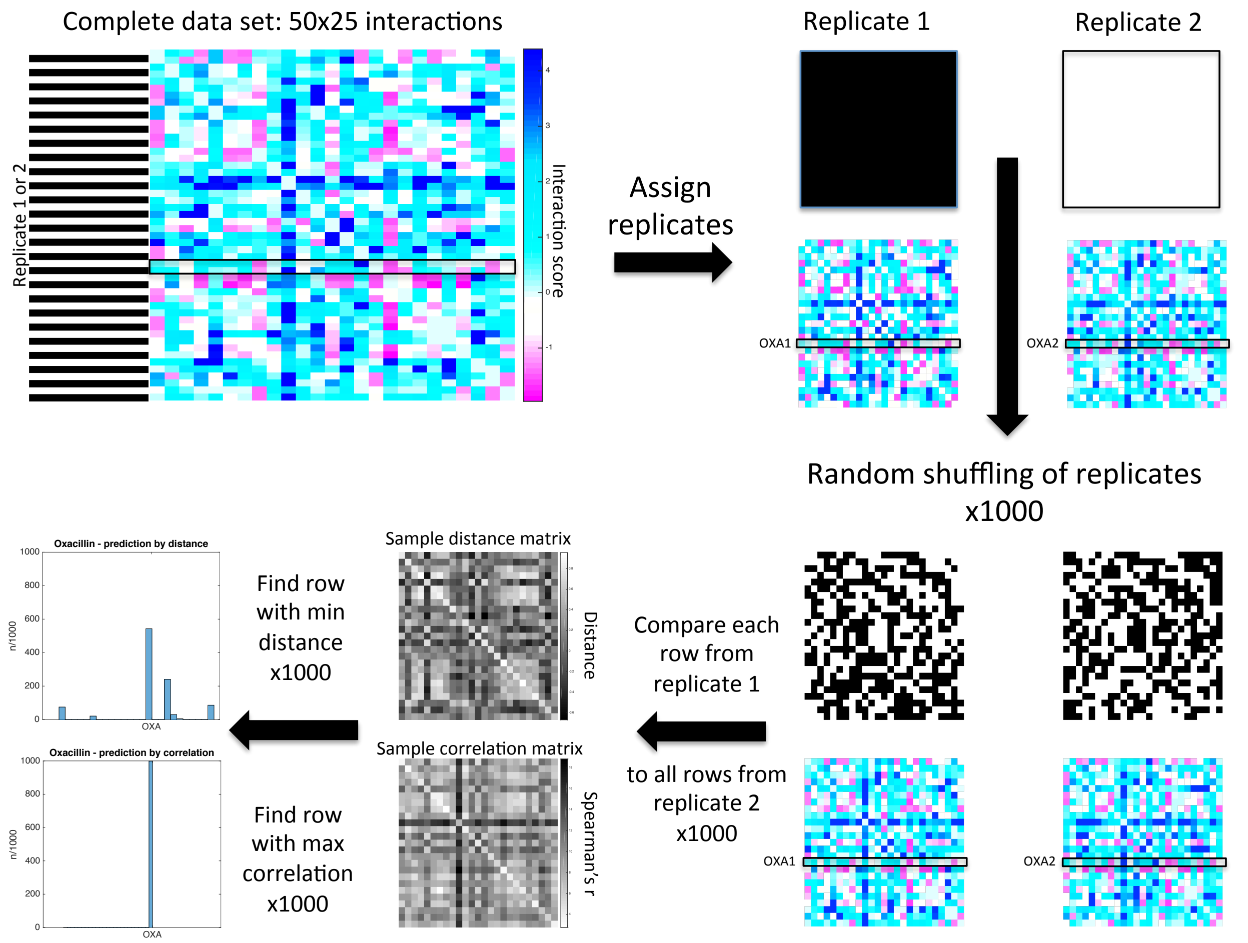

Supplementary Figure S1. *Workflow of drug interaction profile analysis*
The entire matrix of drug interactions (50x25 for the literature set) was assigned to replicate 1 or 2. To eliminate systematic bias, the replicates were randomized. This translates to 2 sets of matrices of 25 query drugs x 25 array drugs x 1000 randomizations for the literature set. The Euclidean distance from each row of data from set 1 to all other rows from set 2 was iteratively determined for all 1000 randomizations. A correct identification of the query drug was considered when the minimum Euclidean distance between each row in set 1 corresponded to the same row in set 2 (for example, oxacillin replicate 1 'OXA1' to oxacillin replicate 2 'OXA2'). The same randomized datasets were assessed for the Spearman's correlation between each row of set 1 and all rows in set 2. In this setup, a correct identification of the query drug was considered when the maximum correlation between each row in set 1 corresponded to the same row in set 2. Oxacillin was nearly always identified by its drug interaction similarity by the correlation metric, and was identified in the majority of cases by the distance metric.
